# Discriminative Analysis of Parkinson’s Disease Based on Whole-Brain Functional Connectivity

**DOI:** 10.1371/journal.pone.0124153

**Published:** 2015-04-17

**Authors:** Yongbin Chen, Wanqun Yang, Jinyi Long, Yuhu Zhang, Jieying Feng, Yuanqing Li, Biao Huang

**Affiliations:** 1 Center for Brain Computer Interfaces and Brain Information Processing, South China University of Technology, Guangzhou, China; 2 Department of Radiology, Guangdong Academy of Medical Sciences, Guangdong General Hospital, Guangzhou, Guangdong, China; 3 Department of Neurology, Guangdong Academy of Medical Sciences, Guangdong General Hospital, Guangzhou, Guangdong, China; Wake Forest School of Medicine, UNITED STATES

## Abstract

Recently, there has been an increasing emphasis on applications of pattern recognition and neuroimaging techniques in the effective and accurate diagnosis of psychiatric or neurological disorders. In the present study, we investigated the whole-brain resting-state functional connectivity patterns of Parkinson's disease (PD), which are expected to provide additional information for the clinical diagnosis and treatment of this disease. First, we computed the functional connectivity between each pair of 116 regions of interest derived from a prior atlas. The most discriminative features based on Kendall tau correlation coefficient were then selected. A support vector machine classifier was employed to classify 21 PD patients with 26 demographically matched healthy controls. This method achieved a classification accuracy of 93.62% using leave-one-out cross-validation, with a sensitivity of 90.47% and a specificity of 96.15%. The majority of the most discriminative functional connections were located within or across the default mode, cingulo-opercular and frontal-parietal networks and the cerebellum. These disease-related resting-state network alterations might play important roles in the pathophysiology of this disease. Our results suggest that analyses of whole-brain resting-state functional connectivity patterns have the potential to improve the clinical diagnosis and treatment evaluation of PD.

## Introduction

Parkinson’s disease (PD) is a progressive neurodegenerative disorder that manifests principally as resting tremor, rigidity, akinesia and postural instability [1]. PD is more common in people over age 50 and has a profound impact on the quality of patients’ lives. Traditionally, the diagnosis of PD depends on the observation of symptoms and longitudinal courses, whereas modern diagnoses of such neurological disorders require objective neurological measures. To improve the level of clinical intervention, it is crucial to identify valid and objective biomarkers to distinguish patients with PD from healthy controls.

In recent years, there has been growing interest in exploring the brain variance between patients with neurological or psychiatric disorders such as major depression, schizophrenia and Alzheimer’s disease and healthy controls based on neuroimaging data [2–4]. Early studies mainly determined the statistical group differences by conventional univariate methods, e.g., voxel-based analyses, which separately analyze the alterations of each position of the brain and might ignore potential information or spatial correlations in the data [5]. Therefore, these conventional methods cannot provide higher sensitivity and might not be optimally suited for investigating the diagnostic separation between patients and healthy controls. Recently, there has been an increasing emphasis on the application of pattern recognition techniques such as neural nets, linear discriminant analysis (LDA) and support vector machines (SVMs) in the effective and accurate diagnosis of psychiatric disorders [6–10]. Several studies have shown that these multivariate pattern analyses (MVPA) are capable of extracting stable structural or functional patterns from neuroimaging data and may potentially be useful for identifying significant neuroimaging-based biomarkers because these analyses take into account the pattern information of multiple variables [11].

PD is associated with the degeneration of dopaminergic nigrostriatal neurons with consequent dysfunction of the cortico-striatal-thalamic loops [12]. Investigation of the functional connectivity within these loops in the resting state is fundamental for understanding the pathophysiology of this disease. Thus, functional connectivity research on PD based on functional magnetic resonance imaging (fMRI) data has attracted considerable attention in recent years. For example, Yu and colleagues [13] found enhanced functional connectivity between the putamen and supplementary motor area (SMA) in PD patients compared with normal controls using either the putamen or the SMA as the “seed region”. However, the changes in functional connectivity are not restricted to the cortico-striatal-thalamic loops, and other regions may be implicated. For example, the striatum exhibited reduced connectivity with the midbrain, pons and cerebellum [14]. Decreased connectivity between the inferior parietal lobule and the dentate nucleus has also been observed in patients with PD [15]. Luo and colleagues [16] found that PD patients showed significantly reduced putamen connectivity with the mesolimbic regions, especially in the amygdala, hippocampus, olfactory area and posterior rectus. The aforementioned findings have demonstrated that functional connectivity abnormalities are widely distributed throughout the entire brain rather than restricted to a few specific brain regions. However, early functional connectivity studies on PD mainly focused on certain brain regions such as the putamen, thalamus or SMA. To date, there has been little focus on examining the functional connectivity throughout the entire brain. It is unknown whether certain unique patterns of functional connectivity associated with PD exist as potential biomarkers that could provide additional information for the clinical diagnosis and treatment of this disease.

Whole-brain functional connectivity analysis based on MVPA might ensure the optimal use of the wealth of information contained in neuroimaging data to identify any neuroimaging-based biomarkers that could be potentially related to different neurological or psychiatric disorders. This type of analysis has been used to discriminate psychiatric disorders including schizophrenia and major depression and has achieved a rather high classification accuracy [10, 17]. In this present study, we focused on the PD discrimination problem using whole-brain functional connectivity. Altered functional connections were observed within or across the default mode, cingulo-opercular and frontal-parietal networks as well as the cerebellum. These alterations may be useful for understanding the underlying neural mechanisms of this disease. Additionally, our results showed that the MVPA method has the potential to improve the clinical diagnosis and treatment evaluation of PD.

## Materials and Methods

### Ethics Statement

This study was approved by the Medical Ethics Committee of Guangdong General Hospital which complies with the Code of Ethics of the World Medical Association (Declaration of Helsinki). Written informed consent was obtained from all patients and controls prior to the MRI examinations. Each patient who took part in this study was diagnosed at an mild or moderate stage (Hoehn & Yahr I-III) and had the ability to sign the consent himself/herself. They were informed of their right to discontinue participation at any time. All potential participants who declined to participate or otherwise did not participate were eligible for treatment (if applicable) and were not disadvantaged in any other way by not participating in the study.

### Participants

The participants included 21 patients (10 males and 11 females; mean age, 58.3 years) who were diagnosed with PD and 26 age- and sex-matched healthy subjects (10 males and 16 females; mean age, 61.3 years). The participants were abstinent from caffeine, nicotine and alcohol prior to scanning. The patients with PD were diagnosed by an experienced neurologist based on the UK Parkinson’s Disease Society Brain Bank Clinical Diagnostic Criteria. Anti-parkinsonian medication was terminated at least 12 hours prior to the imaging scans (OFF medication state). The scores obtained from the Unified Parkinson’s Disease Rating Scale (UPDRS) were assessed for all patients prior to scanning. During the scanning, no moderate-severe head tremor was observed for each patient. The demographic information for the patient and control samples are shown in Table 1.

**Table 1:** Demographic information for the patient and control samples.

**Measure**	**PD (n = 21)**		**HC (n = 26)**		**Statistics**
	**Mean**	**SD**	**Mean**	**SD**	**P-value**
Age (years)	58.3	11.1	61.3	10.1	0.32^*a*^
Gender (male)	10	n.a.	10	n.a.	0.53^*b*^
Illness duration (years)	3.2	3.2	n.a.	n.a.	n.a.
UPDRS	29.8	9.3	n.a.	n.a.	n.a.

Note: PD = Parkinson’s disease, HC = healthy controls, UPDRS = unified Parkinson’s disease rating scale, n.a. = not applicable,

^*a*^Two-sample t-test,

^*b*^Pearson Chi-square test.

### Data acquisition

Brain MRIs were performed on all subjects using the same scanning protocol on a 3.0-T MR scanner (Signa Excite II HD, GE Healthcare, Milwaukee, WI) equipped with a standard 8-channel head coil. Foam padding and earplugs were used to limit head movement and reduce scanner noise. High-resolution T1-weighted structural images were acquired for each subject. The sequence parameters were as follows: rapid interference phase gradient echo flip recovery pulse sequence (FSPGRIR), FA = 13°, FOV = 240 × 240 mm^2^, matrix size = 256 × 256, and slice thickness = 1 mm. These parameters resulted in a voxel size of 0.94 × 0.94 × 1 mm^3^, and 146 sagittal slices were acquired that covered the whole brain. Whole-brain coverage resting-state fMRI data were obtained using gradient-echo echo-planar T2*-weighted imaging (EPI) with the following parameters: 30 slices (in ascending noninterleaved order, parallel to the anterior commissure(AC)—posterior commissure (PC) line), TR = 2000 ms, TE = 30 ms, FA = 80°, FOV = 240 × 240 mm^2^, matrix size = 64 × 64, and slice thickness = 5 mm (no gap). During the data acquisition, individuals were instructed to keep their eyes closed but not to fall asleep, to relax their minds, and to move as little as possible. The scan time for the resting-state fMRI was 372 s, and 186 volumes were obtained.

### Data preprocessing

All resting-state functional MRI images were preprocessed using the Statistical Parametric Mapping (SPM8, http://www.fil.ion.ucl.ac.uk/spm) and Data Processing Assistant for Resting-State fMRI (DPARSF) programs [18]. For each subject, the first 5 volumes of the scanned data were discarded because of the instability of the initial MRI signal and the participants’ adaptation to the circumstance, leaving 181 volumes in total. The remaining images were then corrected for the within-scan acquisition time differences between slices and further realigned to the first volumes to correct for interscan head motions. The motion-corrected functional volumes were spatially normalized to the standard echo planar imaging template in the Montreal Neurological Institute space and then re-sampled to 3-mm cubic voxels. Subsequently, the resulting images were detrended to abandon the linear trend, and then temporally band-pass-filtered (0.01–0.08 Hz) to reduce the effect of the low-frequency drifts and high-frequency physiological noise [19, 20]. Finally, each functional time series was further corrected by regressing out nuisance covariates such as the global signal, white matter signal, cerebrospinal fluid signal and six motion parameters. These nuisance signals are typically adjusted in resting-state functional connectivity studies because they reflect global signal fluctuations of nonneuronal origin (e.g., physiological artifacts associated with cardiac and respiratory cycles, CSF motion and scanner drift). We then calculated the functional connectivity as described below.

### Feature extraction

Functional connectivity, a measure of the correlation strength of two activity time series of anatomically separated brain regions, was used as a classification feature. First, the individual volume was partitioned into 116 ROIs according to the automated anatomical labelling atlas [21]. The atlas divides the cerebrum into 90 regions (45 in each hemisphere) and divides the cerebellum into 26 regions (nine in each cerebellar hemisphere and eight in the vermis). Mean time series for each region were then extracted by averaging the time series within this region using the Resting-State fMRI Data Analysis Toolkit (REST, http://rest.restfmri.net) [22]. We evaluated the functional connectivity between each pair of regions using the Pearson correlation coefficient and obtained (116 × 115) / 2 = 6670 resting-state functional connections for each subject as the feature space for classification.

### Feature selection and classification

Because of our limited number of samples, the leave-one-out cross-validation (LOOCV) method was employed to estimate the performance of the classifier. In each fold of the cross-validation, we selected one sample as testing data and used the remainder as training data. Based on the training data, the discriminative power of a given feature was obtained by calculating its relevance to classification. We then selected features having the greatest discriminative ability as the final feature set and used an SVM classifier to solve the classification problem. The cross-validation was repeated such that each sample was used once as the testing data. In the following section, we explain the process of a fold in detail.

**Feature selection**. In case of low sample size data, high dimensionality might lead to strong side-effects that can significantly bias the estimated performance of the classifier [23]. So dimension reduction is usually performed prior to classification. In addition, the extracted features included the generalization of “noise”, we needed to select features with the most discriminative ability. Given that some features are less effective or are irrelevant or redundant for classification, the selection of a small set of features with the greatest discriminative power will reduce the data dimension, improve the final classification performance and expedite the computation [11, 24]. Here, we used the Kendall tau rank correlation coefficient to measure the discriminative power of each feature [10]. Suppose there are m healthy controls and n patients in the training data. Let x_*ij*_ denote the functional connectivity i of the jth sample and y_*j*_ denote the class label of this sample (+1 for controls and -1 for patients). The Kendall tau rank correlation coefficient of the functional connectivity i can be defined as:

$$\begin{array}{c} \tau_{i}=\frac{n_{c}-n_{d}}{m\times n} \end{array}$$ (1)

where n_*c*_ and n_*d*_ are the numbers of concordant and discordant pairs, respectively. For a pair of samples, one from the controls that denotes {x_*ij*_ y_*j*_} and one from the patients that denotes {x_*ik*_, y_*k*_}, it is a concordant pair when

$$\begin{array}{c} sgn\left(x_{ij}-x_{ik}\right)=sgn\left(y_{j}-y_{k}\right) \end{array}$$ (2)

where *sgn*() is a signum function. Correspondingly, it is a discordant pair when

$$\begin{array}{c} sgn\left(x_{ij}-x_{ik}\right)=-sgn\left(y_{j}-y_{k}\right) \end{array}$$ (3)

Here, we only considered the relationships between two samples that belonged to different groups, and thus the total number of sample pairs was m × n. Obviously, a positive or negative *τ*_*i*_ indicates that the corresponding functional connectivity exhibited a decrease or increase, respectively, in the patients compared with the healthy controls. The discriminative power was defined as the absolute value of the Kendall tau rank correlation coefficient. We then selected k features with the greatest discriminative powers as the final feature set for classification.

The parameter k can be chosen using a parameter search method. In each LOOCV fold, we conducted a new LOOCV using the training data (i.e., using one sample from the training data as new testing data and the others of the training data as new training data) for various values of k. The value of k yielding the best cross-validation accuracy of the training data was chosen. Note that the parameter search was performed on only training data, not the whole data, using the new LOOCV. The computation burden of this parameter search method is excessive, and thus we can simply set k as a constant based on experience, as implemented in many previous studies on the discrimination of psychiatric disorders using whole-brain functional connectivity [10, 17].

**Classification**. In this study, we used an SVM with linear kernel function to construct the classifier. The SVM algorithm was developed using MATLAB (The Math Works, Natwick, MA) and LIBSVM (http://www.csie.ntu.edu.tw/cjlin/libsvm/). In each fold of the LOOCV, we used the selected features having the greatest discriminative powers in the training data with their labels to train the linear classifier with the default setting of the error penalty parameter c = 1. We then applied the SVM to the selected testing data features to determine whether each sample was a patient or a healthy control.

After the LOOCV, we used the generalization rate (i.e., the proportion of all subjects who were correctly classified), sensitivity (i.e., the proportion of the patients who were correctly classified) and specificity (i.e., the proportion of the controls who were correctly classified) to quantify the performance of the classifier. Because different training data were used in each fold of the cross-validation, the final classification feature set after feature selection differed from fold to fold. Consensus functional connectivity was denoted as the functional connectivity that appeared in the final feature set of each fold, and its discriminative power was defined as the average of its discriminative powers across all folds. The region weight, reflecting the significance of identifying PD patients, was defined as the number of consensus functional connections that connected to this brain region [24].

### Permutation tests

In this study, we used a non-parametric permutation test to assess the statistical significance of the classification accuracy [25, 26]. In permutation testing, we randomly permuted the class labels of the training data and performed the above cross-validation procedure 1,000 times. It was assumed that a classifier could reliably learn the relationship between the data and the labels when the generalization rate *GR*_0_ obtained by the classifier that was trained on the real class labels exceeded 95% of the generalization rate obtained using randomly relabeled class labels.

### Head motion analysis

It is known that head motion has a confounding effect on functional connectivity analysis. In order to verify whether the observed group differences were caused by differences in head motion, we investigated the influence of head motion on the measurement of functional connectivity. Four parameters were used to quantify the extent of head motion:

1. **Maximum Motion** was the maximum absolute translation of brain volume as compared to the previous volume.
2. **Mean Motion** represented the mean absolute translation of each brain volume as compared to the previous volume.
3. **Number of Movements** was computed as the number of relative translations greater than 0.1mm between adjacent volumes.
4. **Rotation** was the average of the absolute value of the Euler angle of the rotation of each brain volume as compared to the previous volume. The Euler angle can be computed using the rotation parameters around the three axes:
  $$\begin{array}{c} \theta =arccos(\frac{cos(\alpha )cos(\beta )+cos(\alpha )cos(\gamma )+cos(\beta )cos(\gamma )+sin(\alpha )sin(\beta )sin(\gamma )-1}{2}) \end{array}$$ (4)
  where *α*, *β* and *γ* are three rotation parameters [27].

A group-wise comparison of head-motion was carried out by a two-sample t-test. Furthermore, we tested if the connectivity strength was correlated to any of the four parameters. Specifically, we selected functional connections showing significant group differences according to a two-sample t-test (*α* = 0.001) and correlated each of the four head motion parameters to the connectivity strength of the selected functional connections.

## Results

### Classification results

**Fig 1:**
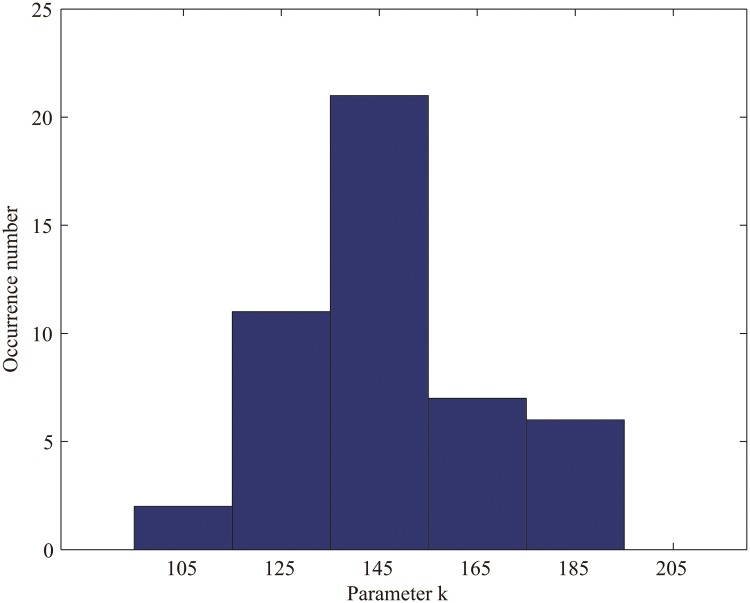
The distribution of numbers of selected features identified using the parameter search method in each fold.

**Fig 2:**
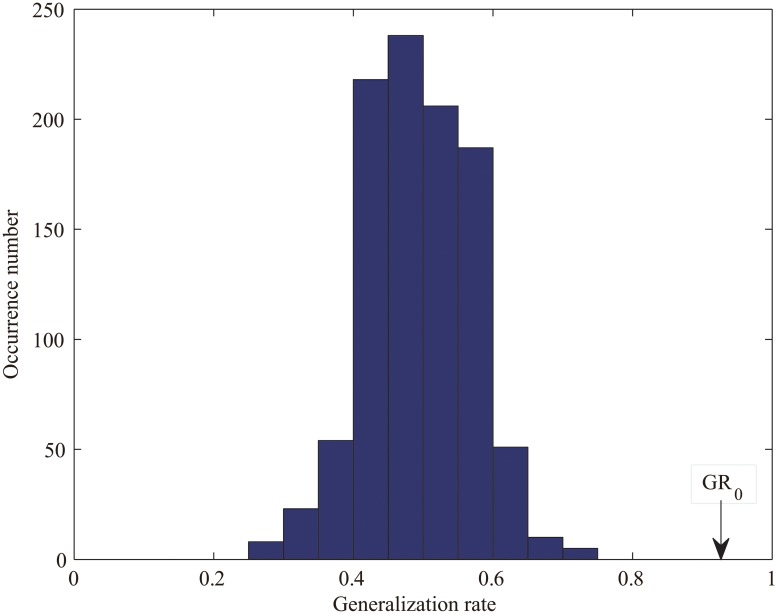
The permutation distribution of the generation rates (1,000 repetitions) when selecting the 150 most discriminating features: the x- and y-labels represent the generalization rate and occurrence number, respectively. *GR*_0_ is the generation rate obtained using the real class labels.

Identifying the number of selected features k using the parameter search method, the classification accuracy for discriminating the individuals as either controls or patients was 95.74% (95.24% sensitivity; 96.15% specificity). The values of the parameter k involved in the classification in each cross-validation fold fell around 145 (Fig 1), and the mean of these values was 149.15. Thus, we simply fixed the parameter k at 150 in each fold, and the classifier achieved an accuracy of 93.62% (permutation test, *p* < 0.001; 90.47% sensitivity; 96.15% specificity). From now on, all the results presented below are based on the selection of a constant parameter k of 150. The permutation distribution of the generation rates is shown in Fig 2. Using the generalization rate as the statistic, the results shown in Fig 2 demonstrate that the classifier learned the relationship between the data and the labels with a probability of being wrong of less than 0.001.

### Region weights and consensus functional connections

**Fig 3:**
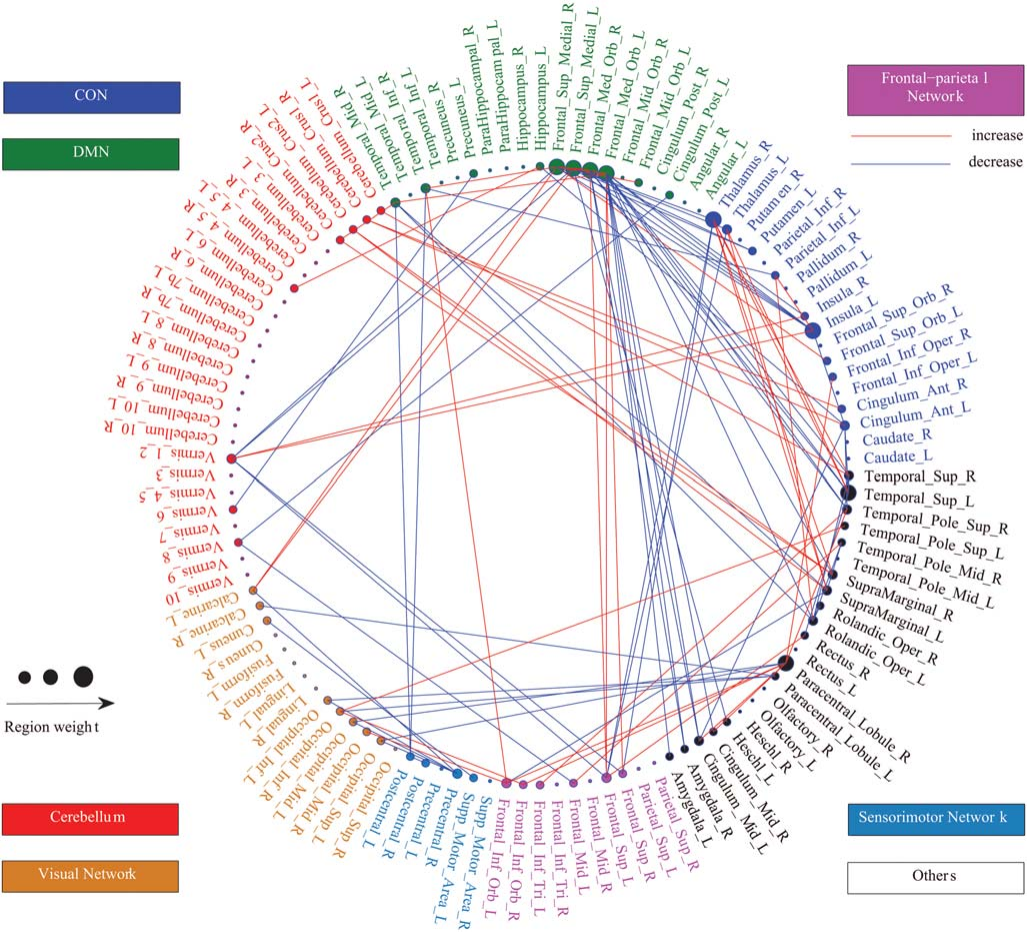
Region weights and the distribution of the 105 consensus functional connections. Regions are color-coded by category (CON, blue; DMN, green; cerebellum, red; visual network, brown; sensorimotor network, cyan; frontal-parietal network, rose; and others, black) and size-coded by weight. The line colors representing the change directions of the consensus functional connections in the patients are red for increases and blue for decreases.

**Fig 4:**
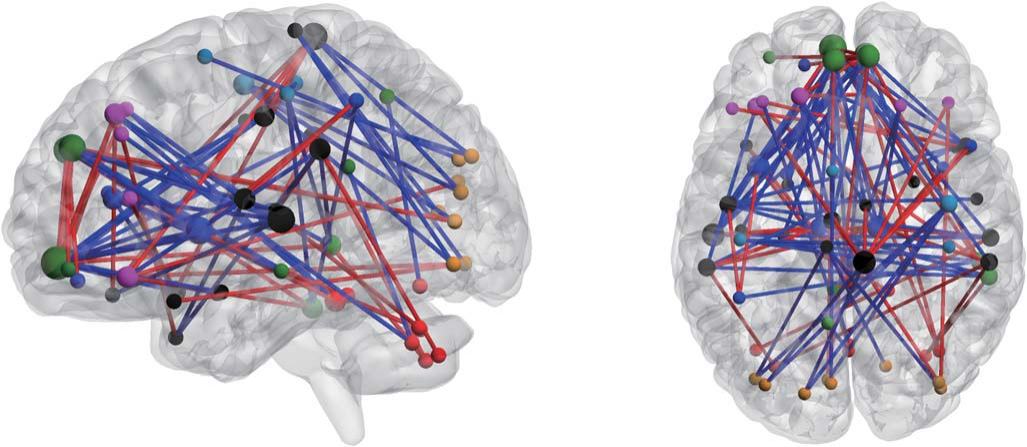
Consensus functional connections demonstrated in the left and top view. Regions are color-coded by category and size-coded by weight as in Fig 3. Red lines represent increased functional connections, and blue lines represent decreased functional connections.

To present the findings in a clear and concise manner, region weights and consensus functional connections are displayed in a circle graph (Fig 3) using a MATLAB tool developed by ourselves, and projected to a surface rendering of a human brain (Fig 4) using the software BrainNet Viewer [28]. Regions are color-coded by category and size-coded by weight. For the patients, the line colors representing the changed directions of the relative consensus functional connections are red for decreases and blue for increases. In this investigation, 105 consensus functional connections were obtained from each fold of the LOOCV, and 58.10% of these connections decreased in the patients compared with the healthy controls. The brain regions related to the consensus functional connectivity were primarily located within the following areas: (i) the default mode network (DMN). mainly containing the bilateral superior frontal gyrus (medial), bilateral superior frontal gyrus (medial orbital), right middle temporal gyrus and right inferior temporal gyrus; (ii) the control network, which can be divided into two independent control networks, the cingulo-opercular network (CON) and the frontal-parietal network [29, 30]. comprising the bilateral thalamus, bilateral insula, bilateral anterior cingulate, bilateral superior frontal gyrus (dorsolateral) and bilateral inferior frontal gyrus (orbital part); and (iii) the cerebellum. In addition, several brain regions also exhibited greater weights than others, ie. the bilateral superior temporal gyrus, right temporal pole: superior temporal gyrus, bilateral supramarginal gyrus, left rolandic operculum, right paracentral lobule and left median cingulate.

### Influence of head motion

The head motion parameters are shown in Table 2. According to a two-sample t-test, no significant difference between the PD patients and healthy controls was found (*p* > 0.05). Furthermore, among the 778 selected functional connections showing significant group differences (*α* = 0.001), only one had a significant correlation (*α* = 0.01) with the motion parameter of the number of movements. Therefore, we concluded that the functional connectivity differences could not be explained by head motion.

**Table 2:** Head motion parameters.

**Quantity**	**PD (n = 21)**		**HC (n = 26)**		**Statistics**	
	**Mean**	**SD**	**Mean**	**SD**	**P-value**	**Significant**
Maximum Motion (mm)	0.1205	0.0879	0.1267	0.0938	0.8179	0
Mean Motion (mm)	0.0704	0.0430	0.0738	0.0395	0.7780	0
Number of Movements	35.1905	40.9971	42.1538	41.1803	0.5665	1
Rotation (*radians* × 1000)	0.7329	0.4152	0.6985	0.3211	0.7504	0

Note: Maximum motion, mean motion, number of movements and rotation for patients (PD) and healthy controls (HC). The p-values are quoted according to a two-sample t-test. We also listed the number of functional connections which significantly correlated to each of the motion parameters.

## Discussion

In this present study, whole-brain functional connectivity was used as a classification feature to discriminate PD patients from healthy controls. The classification performance was validated using LOOCV, and our method achieved an excellent classification accuracy of 93.62%. The majority of the altered functional connections with high discriminative power were located within or across the default mode, cingulo-opercular and frontal-parietal networks and the cerebellum.

### Altered resting-state networks

#### Default mode network

The default mode network is defined as a network that is active at rest and that shows reduced activation during cognitively complex tasks [31]. In this study, the bilateral superior frontal gyrus (medial) and bilateral superior frontal gyrus (medial orbital) exhibited large region weights. The altered functional connections of the above regions may be related to the cognitive decline in patients with PD. DMN abnormalities have been linked to cognitive deficits in a number of neurologic and psychiatric disorders, such as Alzheimer’s disease, autism and dementia [32]. Although PD has traditionally been defined as a motor disorder, it can also result in widespread cognitive impairments, even in the early stages of the disease [33, 34]. Some previous studies have demonstrated altered DMN functional connections in PD patients. For example, Tessitore et al. [35] found decreased functional connectivity in the right medial temporal lobe and bilateral inferior parietal cortex in cognitively unimpaired patients with PD. Although the patients with PD were cognitively unimpaired, the decreased DMN connectivity significantly correlated with cognitive parameters such as memory test results and visuospatial scores. Liu and colleagues [15] found that a set of regions consistent with the DMN showed disrupted connectivity with the dentate nucleus. In our study, compared with those of the healthy controls, most of the DMN regions in the PD patients showed decreased functional connections with other brain regions, in accordance with previous studies. This finding may stem from the reduced thalamic outflows with impaired input or output information to or from the DMN cortices, which may be one explanation for the underlying pathophysiology of these cognitive deficits. Normal cognitive function requires interaction between the DMN and other brain regions, and disrupted interactions may account for the cognitive impairment in PD. Furthermore, we found that the PD patients showed increased connections within the default mode network including the inferior and middle temporal gyri. A possible explanation for this finding is that the abnormal connections within the DMN compensate for the reduced thalamic outflows to and from the DMN as just mentioned.

#### Control network

Classical accounts of the pathophysiology of PD have emphasized the degeneration of dopaminergic nigrostriatal neurons with the consequent dysfunction of the striatum-thalamo-cortical loops. Such dysfunction is believed to lead to motor features including tremor, akinesia and rigor [1, 12]. As one of the important hubs within these loops, the thalamus exhibited great region weight in this present study. Some recent studies have found decreased connections of the thalamus in patients with PD. For example, Hacker and colleagues [14] found markedly lower striatal correlations with the thalamus in PD patients compared with healthy controls. Wu et al. [36] found that the substantia nigra pars compacta had decreased connectivity with the thalamus. In our study, significantly decreased connections of the thalamus with the superior frontal gyrus (dorsolateral) in the frontal-parietal network were observed. This may be one explanation for the executive dysfunction and processing speed deficiencies observed in the early stages of PD. Additionally, the increased functional connections between the thalamus and the superior temporal gyrus and supramarginal gyrus were unexpected. The neural mechanism underlying these increased connections is not understood.

Furthermore, patients with PD experience a range of non-motor symptoms including behavioral changes and somatosensory and autonomic disturbances. As a key region of somatosensory and autonomic information integration, the insula has been found to be extensively related to somatosensory and autonomic dysfunction in PD. For example, Brefel-Courbon and colleagues [37] found that patients with PD OFF medication experienced lower pain thresholds associated with increased activation in the right insula. However, when ON medication this activation was within the normal range. Papapetropoulos et al. [38] found that there was an association between the severity of Lewy body (marker of PD related neurodegeneration) related pathology in the insula and the presence of orthostatic hypotension in PD patients. In this study, the insula also exhibited large region weight. The abnormal functional connections of the insula may contribute to somatosensory and autonomic disturbances in PD patients.

#### Cerebellum

The cerebellum has been recognized as being important in the coordination of voluntary movement, gait, posture and motor functions, and increasing evidence suggests that the cerebellum may contribute substantially to the clinical symptoms of PD [39, 40]. A number of previous studies have reported pathological and structural changes in the cerebellum in patients with PD [41–44]. In the current study, altered connections were observed between the cerebellum and the regions in the default mode, cingulo-opercular and frontal-parietal networks and other regions such as the supramarginal gyrus. Abnormal cerebellar functional connectivity may reflect these pathological and structural changes in association with PD. In addition, most of the altered connections showed a significant increase in the patients with PD compared with the healthy controls. The nature of the increased connections remains unclear, but one likely explanation is that this phenomenon presents a compensatory effect. It is likely that the increased connections of the cerebellum compensate for the hypofunction in the striato-thalamo-cortical circuit to maintain motor function at a near-normal level [45].

### Informative characteristics for classification

In this study, we used whole-brain functional connectivity as a classification feature to discriminate PD patients from healthy controls. Our method achieved a classification accuracy of 93.62%. Recently, a number of brain imaging studies have attempted to distinguish PD patients from healthy controls [46–49], as shown in Table 3. Although a high classification accuracy of 94.4% was obtained in [49], the imaging method was invasive and not suitable as a routine diagnostic tool. Some non-invasive methods achieved a good classification accuracy of 86.96% using multi-type feature combinations that included gray matter (GM), white matter (WM), cerebrospinal fluid (CSF), the amplitude of low-frequency fluctuations (ALFF), regional homogeneity (ReHo) and regional functional connectivity strength (RFCS), but no studies have achieved the high accuracy level of our classification results [47]. Thus, we believe that whole-brain functional connectivity provides more information for discrimination than do any other characteristics (GM, WM, CSF, ALFF, ReHo and RFCS).

**Table 3:** Existing studies regarding Parkinson’s disease classification.

**Literature**	**Subjects**	**Modality**	**Method**	**Accuracy**
Salvatore et al. [46]	28 patients and 28 healthy controls	sMRI	Feature extraction: PCA, classification: SVM.	83.2%
Long et al. [47]	19 patients and 27 healthy controls	sMRI and fMRI	Feature selection: two-sample t-test on the multi-level characteristics (ALFF, ReHo, RFCS, GM, WM and CSF), classification: SVM.	86.96%
Szewczyk-Krolikowski et al. [48]	32 patients and 19 healthy controls	fMRI	Feature extraction: ICA-derived BG network, classification: a threshold method.	85%
Acton and Newberg [49]	81 patients and 94 healthy controls	SPECT	Classification: Artificial neural network.	94.4%

Note: sMRI = structural magnetic resonance imaging, fMRI = functional magnetic resonance imaging, SPECT = single-photon emission computed tomography, PCA = principal component analysis, SVM = support vector machine, ALFF = amplitude of low-frequency fluctuations, ReHo = regional homogeneity, RFCS = regional functional connectivity strength, GM = gray matter, WM = white matter, CSF = cerebrospinal fluid, ICA = independent component analysis, BG = basal ganglia.

### Limitations and future directions

Although the classification results using resting-state functional connectivity were encouraging, there were a number of limitations in this study. First, we defined the AAL regions as nodes. However the AAL regions are quite large and heterogeneous. Averaging over such large regions may thus not give reliable results [50]. Craddock et al. [51] introduced a data-driven method for generating an ROI atlas by parcellating whole brain resting-state fMRI data into spatially coherent regions. It would be our future work to explore the changes in functional connectivity patterns of PD using such atlases in which the regions are more homogeneous. Second, our methodology of using connectivity network is relatively simple. We just concatenated functional connections into a vector for subsequent feature selection and classification. However, some useful structural information of network, especially global topological information, may be lost. It’s worth noting that there are more advanced ways to extracting features. For example, Jie et al. [52] proposed a classification framework involving the use of a new graph-kernel-based approach to identify accurately the mild cognitive impairment patients from healthy controls. Zeng and colleagues [53] proposed a unsupervised classification framework to distinguish the major depression patients from normal controls, which may be the latest progress in the multivariate classification of brain imaging. It would be our future work to further improve the performances of PD diagnosis using such advanced techniques. Finally, correlation analysis should be performed with patient data in the future to explore whether the characteristic brain functional connectivity changes are associated with various disease symptoms and their severity, such as disease duration, motor impairment (measured using the UPDRS III score), levodopa-equivalent daily dose (LEDD) and single cognitive tests.

## Conclusions

In this study, we used whole-brain functional connectivity as a classification feature to identify PD patients and healthy controls. The performance of this method was very high, yielding an accuracy of 93.62%. This promising classification power suggests that this method may improve the clinical diagnosis of PD. The majority of the most discriminating functional connections were located within or across the default mode, cingulo-opercular and frontal-parietal networks and the cerebellum, thereby indicating that the disease-related resting-state network alterations may play important roles in the pathophysiology of this disorder. Future studies are necessary to investigate the relationship between the characteristic brain functional connectivity of patients and their PD symptoms.
